# Short-Term Regulation of Fc*γ*R-Mediated Phagocytosis by TLRs in Macrophages: Participation of 5-Lipoxygenase Products

**DOI:** 10.1155/2017/2086840

**Published:** 2017-08-15

**Authors:** Carla da S. Pinheiro, Ana Paula T. Monteiro, Fabiano F. Dutra, Marcelo T. Bozza, Marc Peters-Golden, Claudia F. Benjamim, Claudio Canetti

**Affiliations:** ^1^Laboratory of Inflammation, Biophysics Institute Carlos Chagas Filho, Rio de Janeiro, RJ, Brazil; ^2^Department of Immunology, Institute of Microbiology Professor Paulo de Góes, Rio de Janeiro, RJ, Brazil; ^3^Division of Pulmonary and Critical Care Medicine, Department of Internal Medicine, University of Michigan, Ann Arbor, MI 48109, USA; ^4^Laboratory of Immunopharmacology, Biophysics Institute Carlos Chagas Filho, Federal University of Rio de Janeiro, 22541-900 Rio de Janeiro, RJ, Brazil

## Abstract

TLRs recognize a broad spectrum of microorganism molecules, triggering a variety of cellular responses. Among them, phagocytosis is a critical process for host defense. Leukotrienes (LTs), lipid mediators produced from 5-lipoxygenase (5-LO) enzyme, increase Fc*γ*R-mediated phagocytosis. Here, we evaluated the participation of TLR2, TLR3, TLR4, and TLR9 in Fc*γ*R-mediated phagocytosis and whether this process is modulated by LTs. Rat alveolar macrophages (AMs), murine bone marrow-derived macrophages (BMDMs), and peritoneal macrophages (PMs) treated with TLR2, TLR3, and TLR4 agonists, but not TLR9, enhanced IgG-opsonized sheep red blood cell (IgG-sRBC) phagocytosis. Pretreatment of AMs or BMDMs with drugs that block LT synthesis impaired the phagocytosis promoted by TLR ligands, and TLR potentiation was also abrogated in PMs and BMDMs from 5-LO^−/−^ mice. LTB_4_ production induced by IgG engagement was amplified by TLR ligands, while cys-LTs were amplified by activation of TLR2 and TLR4, but not by TLR3. We also noted higher ERK1/2 phosphorylation in IgG-RBC-challenged cells when preincubated with TLR agonists. Furthermore, ERK1/2 inhibition by PD98059 reduced the phagocytic activity evoked by TLR agonists. Together, these data indicate that TLR2, TLR3, and TLR4 ligands, but not TLR9, amplify IgG-mediated phagocytosis by a mechanism which requires LT production and ERK-1/2 pathway activation.

## 1. Introduction

Macrophages are professional phagocytes with key cellular contributions in innate immunity, namely, recognizing, ingesting, and eliminating microbial pathogens [1]. Ingestion via the Fc*γ*R is important not only for antibody-mediated phagocytosis of microbes but also for antigen processing and presentation to T cells [2]. Thereby, Fc*γ*Rs contribute to expanding, sustaining, and regulating immune responses [3]. Fc*γ*Rs are subdivided into 3 types (I–III), which contain immunoreceptor tyrosine-based activation motif (ITAM) or immunoreceptor tyrosine-based inhibition motif (ITIM; Fc*γ*RIIb) sequence whose signal transduction events have been extensively studied [4]. During microbial infection, pathogen-associated molecular patterns (PAMPs), specific structures present in microbial pathogens, can elicit immune response through pattern recognition receptors (PRRs) [5]. Among PRRs, the TLRs recognize a broad spectrum of molecules—carbohydrates, lipids, proteins, and nucleic acids—expressed by different microorganisms [6]. Many TLRs have in their structures a leucine-rich repeat, called the TIR (Toll/IL-1-receptor) domain, which interacts with adapter proteins such as MyD88, TRAM, and TRIF to initiate signaling, leading to NF-*κ*B activation and the expression of proinflammatory cytokines [7–10]. Among TLRs, TLR2 plays an important role in the recognition of peptidoglycan, lipoteichoic acid, and lipoprotein, while TLR4 recognizes LPS, a cell-wall component of gram-negative bacteria [11, 12]. TLR3 recognizes polyinosine-polycytidylic acid (poly I:C), a synthetic double-stranded RNA (dsRNA) which mimics viral dsRNA generated during replication of single-stranded RNA (ssRNA) viruses [13]. On the other hand, TLR9 is a receptor for DNA with an unmethylated CpG-motif (CpG-DNA), abundant in bacterial DNA [14]. Ligation of TLRs has been shown to trigger MyD88-dependent signaling through IRAK-4 and p38, leading to the upregulation of a number of phagocytic gene expression programs [15–17]. Studies have found that TLR2 is involved in the phagocytosis of amyloid *β* peptide by microglia [18] and fungi by macrophages [19] and is involved in the phagocytosis of peptidoglycan, an immune adjuvant derived from bacterial cell walls, by intestinal epithelial cells [20]. It is important to emphasize that the majority of studies have evaluated TLR effects in a time range of 12–24 h because of their recognized dependence on changes in gene expression. By contrast, possible short-term effects independent of gene expression, such as those modulating Fc*γ*R-mediated phagocytosis, have not been examined. Leukotrienes (LTs) are lipid mediators of inflammation derived from the 5-lipoxygenase (5-LO) pathway of arachidonic acid metabolism that can be rapidly synthesized upon cell stimulation [21]. Among a broad range of actions, LTB_4_ is recognized as a potent leukocyte activator and chemoattractant, while cysteinyl-LTs (cys-LTs; LTC_4_, LTD_4_, and LTE_4_) are best recognized for their actions on smooth muscle contraction and microvascular permeability, especially during allergic reactions [22, 23]. Our group has previously demonstrated that both classes of LTs are able to enhance Fc*γ*R-mediated phagocytosis and killing [24, 25].

In the present study, we sought to evaluate the short-term effects of TLR2, TLR3, TLR4, and TLR9 agonists on Fc*γ*R-mediated phagocytosis, as well as the participation of LTs in this interaction. We demonstrated in a variety of macrophage populations that Fc*γ*R-mediated phagocytosis is amplified in a dose- and time-dependent manner by treatment with TLR2, TLR3, and TLR4 agonists, but not by TLR9 ligand. The enhancement in the phagocytosis evoked by TLR ligands was abrogated by the absence or inhibition of 5-LO. This contribution of LTs was explained by the observation that TLRs potentiate Fc*γ*R-mediated LT release as well as the LT-dependent ERK-1/2 phosphorylation. Taken together, our findings reveal that 5-LO metabolites modulate the potentiation by TLRs of Fc*γ*R-mediated phagocytosis in the initial events that occur during microbial engagement in macrophages.

## 2. Material and Methods

### 2.1. Animals

5-LO-deficient (5-LO^−/−^ and 129-Alox5^tm1Fun^) [26] mice and strain-matched WT mice were bred in the Laboratory of Transgenic Animals (BIO-Rio, UFRJ) from breeders obtained from Jackson Laboratories (Bar Harbor, ME). Male Wistar rats were obtained from Oswaldo Cruz Foundation (Fiocruz, Rio de Janeiro, Brazil). All experiments were carried out in compliance with the guidelines of the Institutional Animal Welfare Committee (DFBCICB028).

### 2.2. Materials

TLR ligands—TLR2 (Pam3Cys), TLR3 (poly I:C), TLR4 (LPS), and TLR9 (CpG) agonists—were purchased from InvivoGen (San Diego, CA). Zileuton [N-(1-benzo[b]thien-2-ylethyl)-N-hydroxyure] was obtained from Ono Pharmaceutical (Osaka, Japan) and AA861 from Biomol Research Laboratories (Plymouth Meeting, PA). PD98059 was purchased from Cell Signaling (Danvers, MA). Thioglycollate (Tg), RPMI 1640, and all other chemicals were obtained from Sigma (St. Louis, MO).

### 2.3. Cell Culture

Rat resident alveolar macrophages (AMs) were obtained by lung lavage as previously described [27]. Peritoneal macrophages (PMs) were harvested by peritoneal lavage with cold sterile 0,1% heparin-PBS (pH 7.4) three days after the administration of 1 ml of thioglycollate 3% into the peritoneum. Bone marrow-derived macrophages (BMDMs) were prepared using cells obtained by flushing the tibia and femur of 6- to 12-week-old mice with cold sterile RPMI. The differentiation medium was RPMI supplemented with 20% (vol/vol) heat-inactivated FCS and 30% (vol/vol) L929 cell supernatant. Initially, 5 × 10^6^ bone marrow cells were suspended in 10 ml of differentiation medium and then seeded in 100 mm Petri dishes (BD Biosciences) at 37°C in humidified 5% CO_2_. After 3 d, 10 ml of differentiation medium was added. Finally, at day 6, cells were washed with cold RPMI and suspended and seeded at the required density for all experiments [28]. Cell suspension was enumerated using a hemocytometer, adhered in flat bottom 96- or 24-well plates (0.15 and 1 × 10^6^ cells/well, resp.; BD Biosciences, San Jose, CA), and cultured in DMEM at 37°C in a 5% CO_2_ atmosphere. After 1 h, nonadherent cells were removed by washing. Macrophage monolayers were cultured overnight in DMEM supplemented with 10% heat-inactivated endotoxin-free fetal cow serum (FCS). The cells were washed, and the medium was changed to DMEM without serum 30 min before the treatment with the TLR agonists.

### 2.4. Erythrocyte Opsonization and Microcolorimetric Phagocytosis Assay

Sheep red blood cells (RBCs) were opsonized with a subagglutinating concentration of IgG rabbit antisheep erythrocyte antibody (Cappel Organon Teknika, Durham, NC) as previously described [29]. The phagocytosis of IgG-coated RBCs (IgG-RBCs) was assayed using adherent macrophage monolayers in DMEM medium as described previously [30]. Briefly, rat AMs or mouse PMs or BMDM were cultured in 96-well plates as described and preincubated with TLR agonists for 5–60 min. Following preincubation, opsonized RBCs were added at a target: cell ratio of 40 : 1, and cultures were incubated for an additional 90 min at 37°C. Wells were then washed three times with PBS to remove uningested erythrocytes. Serial dilutions of known amounts of RBCs were added to separate wells to derive a standard curve, and 75 *μ*l of 0.3% SDS in PBS was added to each well for 10 min. Lastly, 50 *μ*l of 3,3′,5,5′-tetramethyl-benzidine dihydrochloride hydrate (TMB) (0,2 mg·ml^−1^) solution was added to each well as a chromogen. Following a 5 min incubation (at room temperature) in the dark, the reaction was stopped with 100 *μ*l H_2_SO_4_ (2 M) and the absorbance was evaluated at 450 nm with an automated reader (SOFTmax, Molecular Devices). The number of RBCs per well was derived from absorbance data at 450 nm using the standard curve made. Independent experiments were performed in septuplet.

### 2.5. Measurement of LTs

PMs were cultured in 24-well plates in DMEM at concentrations of 1 × 10^6^ cells/well as described above. For LT determination, cells were incubated with TLR agonists and then challenged with IgG-RBCs, unopsonized SRBCs, or medium alone for 2 h. Culture supernatants were harvested, and LT levels were quantified by enzyme immunoassay kits according to the manufacturer instructions (Cayman Chemical Company, Ann Arbor, MI).

### 2.6. Immunoblotting

PM monolayers were lysed in 1% Triton X-100 buffer containing 50 mM Tris [tris(hydroxymethyl)aminomethane] (pH 8), 100 mM NaCl, 1 mM Na_3_VO_4_, 1 mM PMSF (phenylmethylsulfonyl fluoride), 50 mM NaF, and 1 *μ*g·ml^−1^ leupeptin. Lysates were separated on SDS 8% polyacrylamide gel electrophoresis (SDS-PAGE) gels. The proteins were transferred to nitrocellulose membranes (Schleicher & Schuell, Keene, NH) overnight at 100 amps (A) and for 3 h at 200 A. Next, the membrane was blocked with 5% fat-free milk in TBST 0.1% for 1 h, washed 3 times, and then probed with antiphosphorylated ERK-1/2 (Cell Signaling) for 1.5 h. After that, the membranes were washed and incubated with a horseradish-peroxidase- (HRP-) conjugated secondary antibody (1 : 10,000; Amersham Pharmacia Biotech, Piscataway, NJ). Phosphorylated bands were visualized using the enhanced chemiluminescence system (ECL, Amersham, Arlington Heights, IL). The membranes were then stripped, blocked, and reprobed with anti-ERK-1/2 or anti-GAPDH for 1 h followed by an incubation with HRP-conjugated secondary antibody (1 : 15,000; Amersham Pharmacia Biotech). The bands were visualized using the ECL system.

### 2.7. Statistical Analysis

The data are reported as a representative graph from 2 or 3 independent experiments. Graphs represent the mean ± SEM and were analyzed with the Prism 4.0 statistical program (GraphPad Software, San Diego, CA). Comparisons were performed with Student's *t*-test as indicated in figure legends. Differences were considered significant if *P* ≤ 0.05. Representative immunoblots of at least 2 independent experiments are depicted, as also stated in the legend of the respective figure.

## 3. Results

### 3.1. TLR Agonists Increase Fc*γ*R-Mediated Phagocytosis

**Figure 1:**
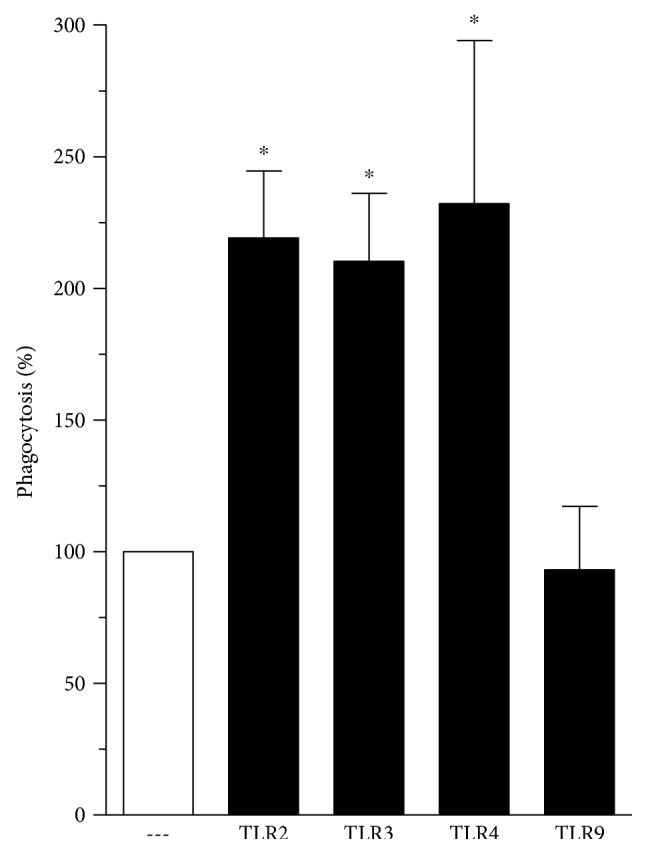
TLR agonists increase Fc*γ*R-mediated phagocytosis. Rat AMs were pretreated in absence or presence of TLR2 (Pam3Cys: 25 *μ*g·ml^−1^), TLR3 (poly I:C: 1 *μ*g·ml^−1^), TLR4 (LPS: 1 *μ*g·ml^−1^), or TLR9 (CpG: 1 *μ*g·ml^−1^) agonists for 1 h and then challenged with RBCs or IgG-RBCs (1 : 40). Results are expressed as the mean ± SEM. Values are presented as the percentage of the IgG-RBC group. The RBC control group was discounted from all groups. ^∗^*P* < 0.05 compared to the IgG-RBC group. The experiment is a representative of three independent experiments performed in heptaplicate.

**Figure 2:**
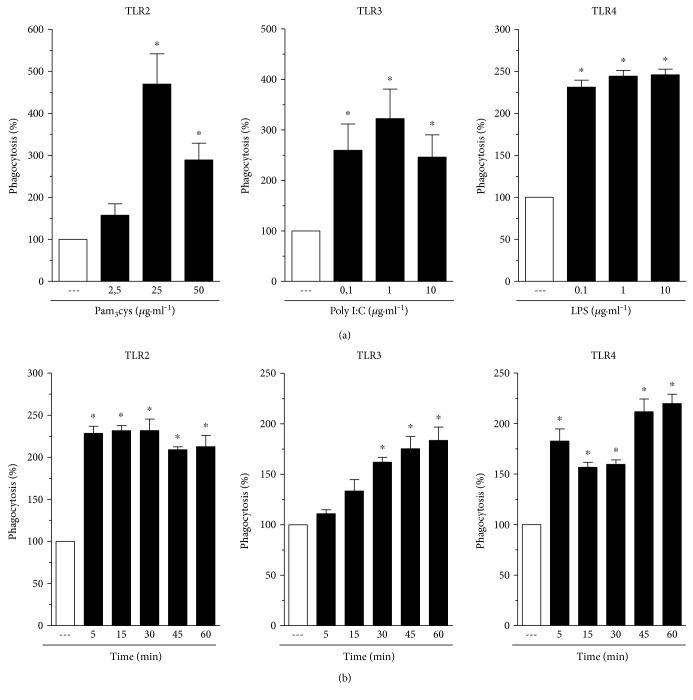
TLR agonists increase IgG-sRBC phagocytosis in a dose- and time-dependent manner. (a) Rat AMs were cultured in the absence or in the presence of TLR2 (Pam3Cys), TLR3 (poly I:C), TLR4 (LPS) agonists at the indicated concentrations for 1 h. (b) The cells were cultured with TLR2 (Pam3Cys: 25 *μ*g·ml^−1^), TLR3 (poly I:C: 1 *μ*g·ml^−1^), or TLR4 (LPS: 1 *μ*g·ml^−1^) at the indicated time periods. In both panels, rat AMs were challenged with RBCs or IgG-RBCs (1 : 40) after TLR agonist stimulation. Results are expressed as the mean ± SEM. Values are presented as the percentage of the IgG-RBC group. The RBC control group was discounted from all groups. ^∗^*P* < 0.05 compared to the IgG-RBC group. The experiment is a representative of three independent experiments performed in heptaplicate.

In 2007, Anand and colleagues demonstrated that treatment of mice PMs, as well as the J774 macrophage cell line, with LPS increased Fc*γ*R-mediated phagocytosis [31]. Initially, we sought to investigate if ligands of TLRs other than TLR4 would also be able to augment Fc*γ*R-mediated phagocytosis; we also examined the kinetics of this effect and its applicability in different macrophage populations. As can be seen in Figure 1(), pretreatment with TLR2 (Pam3Cys: 25 *μ*g·ml^−1^), TLR3 (poly I:C: 1 *μ*g·ml^−1^), and TLR4 (LPS: 1 *μ*g·ml^−1^) agonists for 1 h increased Fc*γ*R-mediated phagocytosis in AMs, while TLR9 agonist (CpG: 1 *μ*g·ml^−1^) treatment did not. The same findings were obtained using BMDM (Figure 2()) and PMs (data not shown) obtained from mice, suggesting that this is a generalized response of macrophages. The lack of effect of the TLR9 agonist on phagocytosis was not attributable to an inadequate/low dose, as the same result was obtained with a dose 10 times higher (data not shown). In order to rule out the possibility of LPS contamination of these other TLR ligands used, we incubated PMs from WT and TLR2^−/−^ mice with Pam3Cys and evaluated phagocytosis. As expected, PM stimulation with Pam3Cys augmented phagocytosis in WT cells, but not in TLR2^−/−^ cells (data not shown).

### 3.2. Dose-Dependent Effects of TLR Agonists on Fc*γ*R-Mediated Phagocytosis

We next examined the dose dependency of the ability of agonists of TLR2, TLR3, and TLR4 to increase Fc*γ*R-mediated phagocytosis.  Figure 2(a) (left panel) demonstrates that treatment of AMs with TLR2 agonist (Pam3Cys) showed a concentration-dependent effect of Fc*γ*R-mediated phagocytosis, which reached a maximal effect at 25 *μ*g·ml^−1^. The effect of phagocytosis promoted by TLR3 and TLR4 agonists (poly I:C and LPS, resp.) was of similar magnitude for all three doses tested (Figure 2(a); middle and right panels).

### 3.3. Time-Dependent Effects of TLR Agonists on Fc*γ*R-Mediated Phagocytosis

We next examined the kinetics of this potentiating effect of TLR agonists on Fc*γ*R-mediated phagocytosis. The maximal effect of the TLR2 agonist was seen with pretreatment intervals as short as 5 (Figure 2(b), left panel). TLR4 ligation by LPS also induced an increase in phagocytosis at all time points evaluated, reaching peaks at 5 and 45–60 min of pretreatment (Figure 2(b), right panel). On the other hand, the effects of the TLR3 agonist were time-dependent over the interval from 5 to 60 min, reaching statistical significance at 30 min.

### 3.4. Pharmacological Inhibition or Genetic 5-LO Depletion Affects Phagocytosis Induced by TLR Agonists

**Figure 3:**
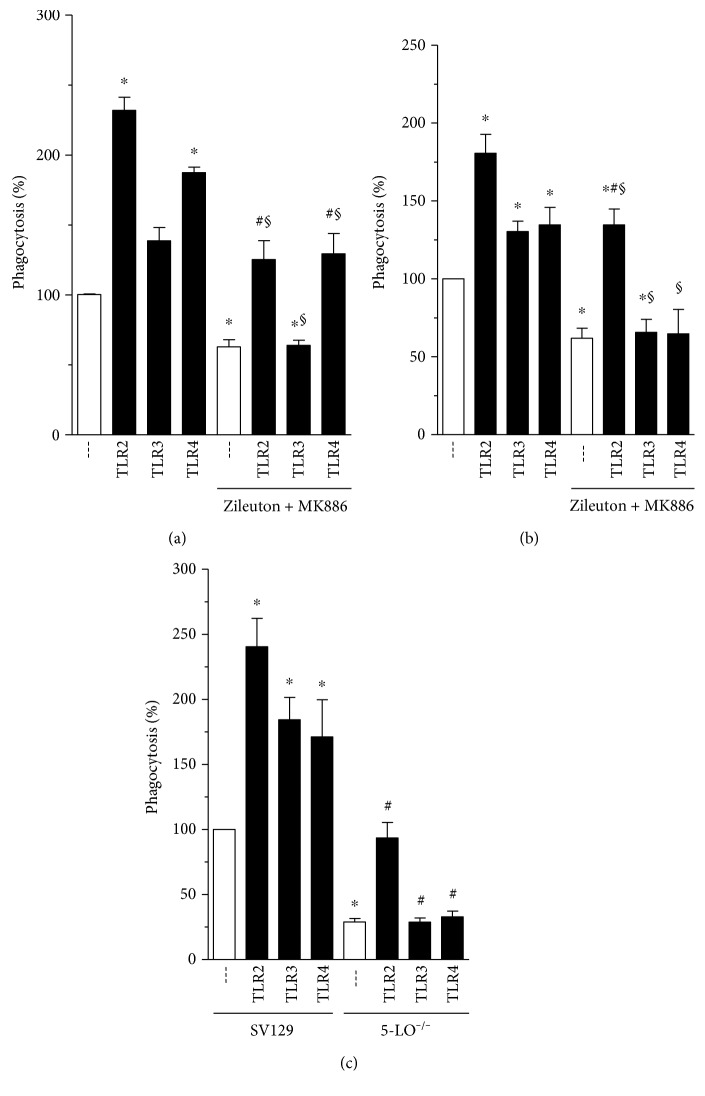
The ability of TLR agonists to enhance Fc*γ*R-mediated phagocytosis is modulated by 5-LO pathway. (a) Rat AMs and (b) mice BMDM were pretreated with medium containing 5-LO inhibitor (zileuton 20 *μ*M) and FLAP inhibitor (MK886, 1 *μ*M) for 20 min. After this period, TLR agonists were added in cell culture, followed by RBCs or IgG-RBCs (1 : 40) in addition. (c) BMDM from WT and 5-LO^−/−^ mice were also pretreated TLR agonists and challenged with RBCs or IgG-RBCs (1 : 40). All experiments used TLR2 (Pam3Cys: 25 *μ*g·ml^−1^), TLR3 (poly I:C: 1 *μ*g·ml^−1^), or TLR4 (LPS: 1 *μ*g·ml^−1^) agonists for 1 h. Results are expressed as the mean ± SEM. Values are presented as the percentage of the IgG-RBC group and the negative control group, and the RBC control group was discounted from all groups. ^∗^*P* < 0.05 compared to the IgG-RBC group; ^#^*P* < 0.05 compared to the IgG-RBC group treated with zileuton + MK886 (a, b) or IgG-RBC 5-LO^−/−^ (c); or ^§^*P* < 0.05 compared to respective the TLR group (without treatment or SV129), determined by Student's *t*-test. The experiment is a representative of three independent experiments performed in heptaplicate.

Although the independent ability of LTs to enhance the FcR-mediated phagocytic process has been established [24, 25, 27], its effect on TLR stimulation is unknown. The first committed step in LT biosynthesis is the 5-lipoxygenation of arachidonate, carried out by the enzyme 5-LO in concert with its helper protein, 5-LO-activating protein (FLAP). As a first test of the role of LTs in this process, we treated rat AMs with a combination of the 5-LO inhibitor zileuton (20 *μ*M) and the FLAP inhibitor MK886 (1 *μ*M). As demonstrated in Figure 3(a), pharmacological inhibition of LT synthesis impaired the augmentation of Fc*γ*R-mediated phagocytosis evoked by TLR2, TLR3, and TLR4 agonists. A similar result was seen in BMDMs (Figure 3(b)). As an alternative approach to interrogating the requirement of LTs in TLR effect on Fc*γ*R-mediated phagocytosis, we utilized BMDMs obtained from 5-LO^−/−^ mice and obtained the same finding (Figure 3(c)). These results, consistent across different macrophage populations and with both genetic and pharmacologic abrogations, indicate that 5-LO activity is essential for phagocytosis mediated by TLR ligands.

### 3.5. TLR Agonists Potentiate LT Secretion following Fc*γ*R Ligation

**Figure 4:**
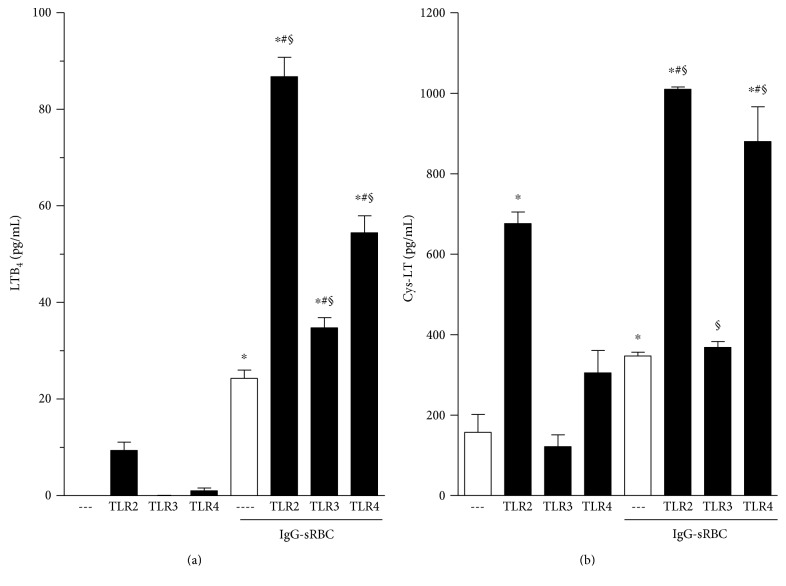
TLR agonists potentiate LTB_4_ (a) and cys-LT (b) secretion following Fc*γ*R ligation. Mouse Tg-elicited PMs were treated with TLR2 (Pam3Cys: 25 *μ*g·ml^−1^), TLR3 (poly I:C: 1 *μ*g·ml^−1^), or TLR4 (LPS: 1 *μ*g·ml^−1^) agonists for 1 h, as indicated in the graphic, and then challenged or not with IgG-RBCs (1 : 40). Results are expressed as the mean ± SEM. Values are presented as the percentage of the IgG-RBC group and the negative control group, and the RBC control group was discounted from all groups. ^∗^*P* < 0.05 compared to cell without IgG-RBC challenge; ^#^*P* < 0.05 compared to the IgG-RBC control and ^§^*P* < 0.05 compared to respective TLR without IgG-RBC challenge determined by Student's *t*-test. The results of one experiment representative of three independent experiments performed in heptaplicate.

In view of the fact that 5-LO deletion or its pharmacological inhibition abrogated the positive effect of TLR agonists on Fc*γ*R-mediated phagocytosis, we investigated whether TLR agonists potentiate LT production in response to Fc*γ*R ligation. As shown in Figure 4(a), neither unstimulated nor TLR agonist-stimulated PMs produce meaningful amounts of LTB_4_. As expected, challenge of PMs with IgG-RBCs, in the absence of TLR agonists, causes the release of LTB_4_. Moreover, LTB_4_ production induced by IgG-RBCs was potentiated in cells pretreated with TLR agonists, especially those engaging TLR2 and TLR4. We also evaluated cys-LT production under the same experimental conditions. As presented in Figure 4(b), a TLR2 agonist was sufficient to elicit cys-LT production by PMs. Cys-LT formation was also induced by the phagocytosis of IgG-coated RBCs, as previously reported [25]. As with LTB_4_ production, cys-LTs synthesis was further augmented in IgG-RBC-challenged PMs treated with TLR2 or TLR4 ligands, but not with a TLR3 agonist (poly I:C; Figure 4(b)).

### 3.6. Fc*γ*R-Mediated ERK-1/2 Activation Is Enhanced by TLRs

**Figure 5:**
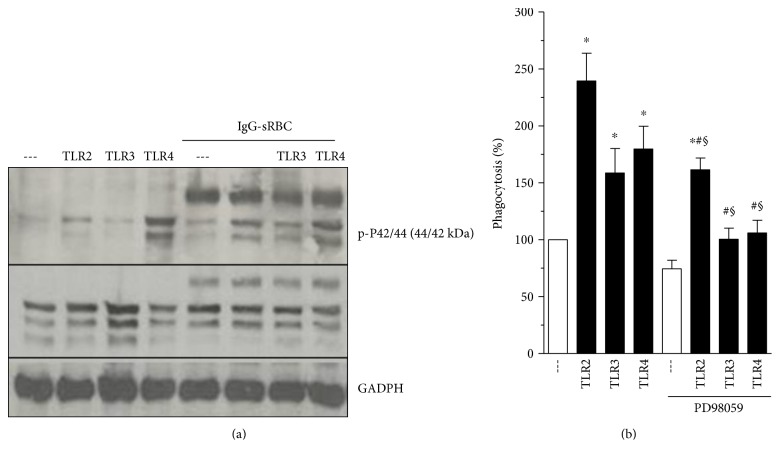
ERK-1/2 inhibition impairs phagocytic process promoted by TLRs. (a) Mouse PMs were pretreated with TLR2 (Pam3Cys: 25 *μ*g·ml^−1^), TLR3 (poly I:C: 1 *μ*g·ml^−1^), or TLR4 (LPS: 1 *μ*g·ml^−1^) agonists for 1 h, and then challenged with IgG-sRBC (1 : 40 ratio) for further 30 min. Incubations were terminated by addition of lysis buffer, and lysates were subjected to immunoblotting as described in Materials and Methods. Immunoblots in the upper panel represent phosphorylated ERK-1/2, middle panel represents total ERK-1/2, and lower panel, the amounts of GAPDH. Immunoblots are representative of two separate experiments. (b) Mouse PMs were pretreated with medium containing MEK inhibitor (PD98059 25 *μ*M) for 20 min and then treated with TLR2 (Pam3Cys: 25 *μ*g·ml^−1^), TLR3 (poly I:C: 1 *μ*g·ml^−1^), or TLR4 (LPS: 1 *μ*g·ml^−1^) agonists for 1 h. The cells were challenged with RBCs or IgG-RBCs (1 : 40). Results are expressed as the mean ± SEM. Values are presented as the percentage of the IgG-RBC group and the negative control group, and the RBC control group was discounted from all groups. ^∗^*P* < 0.05 compared to IgG-RBCs group or ^#^*P* < 0.05 compared to TLR without PD98059. ^§^*P* < 0.05 compared to the respective TLR without IgG-RBC challenge. Statistical analysis was performed using Student's *t*-test. The results of one experiment representative of three independent experiments performed in heptaplicate.

Several studies have identified a role for ERK signaling during phagocytosis, including that signaling which is specifically Fc*γ*R-mediated [32–34]. In order to test if ERK-1/2 is involved in the TLR-mediated enhancement of Fc*γ*R-dependent phagocytosis, PMs were preincubated with TLR agonists and challenged with or without IgG-RBC; after which, ERK-1/2 activation was monitored by immunoblotting with a phospho-specific antibody. As observed in Figure 5(a), in the absence of Fc*γ*R ligation, the treatment of PMs with an agonist for TLR2, but not TLR3, caused a small degree of ERK-1/2 phosphorylation, similar in magnitude to that evoked by Fc*γ*R engagement alone. On the other hand, incubation with a TLR4 agonist (LPS) alone was sufficient to stimulate a more robust degree of ERK-1/2 phosphorylation. Pretreatment with TLR2 or TLR3 agonists prior to challenge with IgG-RBCs resulted in a substantially greater degree of ERK-1/2 phosphorylation than that observed with TLR stimulation or with IgG-RBC challenge alone (Figure 5(a)), suggesting a synergistic effect. No enhancement of ERK-1/2 phosphorylation was noted in cells incubated with LPS and IgG-RBCs than was seen with LPS alone (Figure 5(a)).

### 3.7. ERK-1/2 Inhibition Impairs the Phagocytosis Promoted by TLRs

To evaluate a possible functional role for ERK-1/2 in TLR agonist-induced enhancement of phagocytosis, we tested the effects of PD98059, an inhibitor of MEK, the upstream kinase which activates ERK1/2. As shown in Figure 5(b), the potentiation by TLR agonists of phagocytosis in PMs was significantly decreased in cells treated with PD98059, compared to untreated cells. These findings suggest a convergence of signals generated by TLRs and Fc*γ*R to ERK-1/2 pathway.

## 4. Discussion

There are few data in the literature demonstrating TLR involvement in the phagocytic process [31, 35]. In the present study, we report for the first time that ligands of TLR2, TLR3, and TLR4, but not 9, lead to a rapid enhancement of Fc*γ*R-mediated phagocytosis. This was observed in a variety of rodent macrophage populations, including rat AMs, murine elicited PMs, and murine BMDMs and was mediated by a LT-ERK1/2 axis.

We observed that TLR2 and TLR4 agonists augmented FcR-mediated phagocytosis in as little as 5 min. The augmentation by a TLR3 agonist was only observed after 30 min (Figure 3(b)), likely reflecting the intracellular localization of the TLR3 receptor [36].

It has been established that LTs, lipid mediators derived from the 5-LO pathway, can enhance Fc*γ*R-elicited phagocytosis [24, 25]. Furthermore, 5-LO products are part of FcR signaling, since during FcR-mediated phagocytosis, there occurs the generation of endogenous LT production which is important for the phagocytic process but not essential. Moreover, 5-LO inhibition or genetic deletion causes the reduction of FcR-mediated phagocytosis but does not abolish it. This led us to investigate the possibility of crosstalk between TLRs and LTs in a very short time period. As demonstrated in Figure 2, both pharmacologic and genetic interruptions of LT biosynthesis abrogated the ability of TLR agonists to amplify phagocytosis. It has been reported that loss of TLR4 results in a 50% reduction of cys-LT production after stimulation with HSP70, a protein that recently has been reported to activate TLRs [37]. Supporting our data, Serezani and colleagues either demonstrated that responses dependent on MyD88 (an adaptor protein that mediates signaling through all of the known TLRs, except TLR3) were reduced in mice lacking either 5-LO or BTL1 (LTB_4_ receptor) [38]. Together, these data emphasize an important interplay between TLR, Fc*γ*R, and LT production.

To further understand this interplay, we investigated LT biosynthesis during single and dual stimulation of TLR and Fc*γ*R. Although stimulation with TLR agonists alone was insufficient to result in LTB_4_ production, priming with TLR agonists did significantly increase that production generated in response to challenge with IgG-opsonized targets. Cys-LT production was also enhanced by TLR2 and TLR4 agonist pretreatment. These results demonstrate that PMs primed with TLR agonists, especially TLR2 and TLR4, have a greater LT synthetic capacity following IgG-RBCs challenge. This interplay between TLRs and production of LTs is supported by the recent report that the ability of agonists for TLR1/2, TLR 2/6, TLR3, TLR4, TLR5, and TLR7/8 to promote neutrophil transmigration through epithelial monolayers was markedly blocked by LTB_4_ antagonists [39]. In the present study, we have not delineated the specific 5-LO products responsible for such effect in phagocytosis caused by TLRs.

It is possible that stimulation of TLR triggers LT release, priming cells for Fc*γ*R activation by IgG-RBC and leading to MAPK activation and phagocytosis amplification. However, this sequence of events could differ for distinct TLR ligands. It has been shown that LT production and release depend on MAPK activation [40]. Among the intracellular sites for LT synthesis, it is known that 5-LO resides in the cytoplasm or nucleoplasm of resting cells and can translocate to the nuclear membrane upon activation by Ca^2+^, where it colocalizes with cPLA_2_ and FLAP [41]. The 5-LO C-terminal catalytic domain can be phosphorylated by p38 MAPK, ERKs, and protein kinase A, which modulate 5-LO nuclear localization and LT synthesis [42]. In addition, cytoskeletal rearrangement required for phagocytosis depends on ERK-1/2 activation [43]. In our study, IgG-RBC challenge causes a moderate degree of ERK-1/2 activation which was further enhanced by TLR pretreatment. Another TLR2 ligand (lipoteichoic acid) was demonstrated to induce ERK activation in human smooth muscle cells [44]. Considering an association between TLR ligands and ERK-1/2 activation, the effect of an ERK-1/2 pharmacological inhibitor (PD98059) was tested, and it reduced the potentiating effect of TLRs, pointing to a role for ERK-1/2 pathway activation under these circumstances. Wu and colleagues verified that a pretreatment with LPS induced ERK-1/2 and P38 activation in A549 cells, a phenomenon related to an increase in TLR2 gene expression [45]. Furthermore, Haralambieva and colleagues demonstrated that pretreatment with anti-TLR4 abolished ERK-1/2 activation induced by *Chlamydophila pneumoniae* in human fibroblasts [46].

Based on our findings, we cannot define if LT synthesis and ERK-1/2 activation occur in sequence or in parallel, nor can we define the relative importance of these two events for each TLR agonist tested. Measurement of LTs in the presence of ERK-1/2 inhibitor as well as immunoblots for ERK-1/2 activation under 5-LO inhibition could help to delineate the events generated in this particular scenario of multiple players. Probably, activation by ERK-1/2 could be a result of LTB_4_ release, especially when observed for TLR2 incubation, where ERK-1/2 is activated before IgG-RBC challenge and amplified after Fc*γ* receptor activation. Similar result was obtained by Campos and colleagues, whose study shows that endogenous LTB_4_ contributed to Fc*γ*R-mediated activation of PKC-alpha, ERK-1/2, and PI3K, while endogenous cys-LTs contributes to the activation of PKC-delta, p38, and PI3K [40].

The data presented in the current work does not allow us to indicate the mechanism(s) responsible for the effect of TLR agonists on FcR-mediated phagocytosis. We have previously shown that LTB_4_ amplifies FcR-dependent phagocytosis by affecting Syk activation (increase) and also by a mechanism dependent on Ca^++^. These possibilities could also be true for TLR effects; however, further experiments are necessary to test these hypotheses. Taken this scenario into account, the enhancement of LT production caused by TLR treatment can give a clue in the mechanism of action, supporting the idea that TLR effects are dependent on LT production.

Altogether, our results suggest a positive and rapid enhancement by TLR2, TLR3, and TLR4 ligands of Fc*γ*R-mediated phagocytosis, in a process depending on LT production and/or ERK-1/2 pathway activation. This crosstalk between TLR, LT production, and Fc*γ*R activation, in a very short time point, reflects an integrated and efficient host immune response during the recognition of pathogenic molecules.
